# Metal Decorated B_4_N_4_ Nanocages Quantum Dots for Hydrogen Storage: A Comprehensive Density Functional Theory Approach

**DOI:** 10.3390/nano16090499

**Published:** 2026-04-22

**Authors:** Seyfeddine Rahali, Youghourta Belhocine, Ridha Ben Said, Yusuf Zuntu Abdullah, Tasneem I. Hussein, Bakheit Mustafa

**Affiliations:** 1 Department of Chemistry, College of Science, Qassim University, Buraydah 51452, Saudi Arabia; r.said@qu.edu.sa (R.B.S.);; 2 Laboratory of Catalysis, Bioprocess and Environment, Department of Process Engineering, Faculty of Technology, University of 20 August 1955, Skikda 21000, Algeria; y.belhocine@univ-skikda.dz; 3 Department of Physics, Aydin Adnan Menderes University, Aydin 09010, Turkey; 4 Department of Physics, Faculty of Science, Kaduna State University, Kaduna PMB 2339, Nigeria

**Keywords:** B_4_N_4_ quantum dots, hydrogen storage, metal decoration, DFT, reversible H_2_ uptake

## Abstract

Metal-functionalized boron nitride nanostructures represent promising platforms for lightweight solid-state hydrogen storage. In this work, we perform a comprehensive density functional theory (DFT) investigation of pristine and metal-decorated B_4_N_4_ quantum dots (M = Li, Ti) to evaluate their structural stability, adsorption energetics, and near-ambient storage performance. Pristine B_4_N_4_ is highly stable but interacts weakly with H_2_ (E_ads_ ≈ −0.12 eV), leading to negligible uptake under operating conditions. Li decoration moderately enhances adsorption through charge-induced polarization (E_ads_ ≈ −0.15 eV) but offers limited stabilization beyond the first few molecules. In contrast, Ti decoration fundamentally reshapes the interaction landscape, strengthening electrostatic, polarization, and dispersion contributions and enabling significantly stronger yet reversible H_2_ binding (E_ads_ ≈ −0.36 eV). Sequential adsorption calculations predict maximum theoretical capacities of 14, 18, and 20 H_2_ molecules for pristine, Li-, and Ti-decorated systems, respectively. Grand canonical thermodynamics show that Ti–B_4_N_4_ retains nearly its full loading at 30 bar and 298 K, while pristine and Li-decorated clusters store only negligible amounts. Under desorption conditions (3 bar, 373 K), Ti–B_4_N_4_ releases most of its stored hydrogen, yielding an exceptional reversible capacity of 15.1 wt%. Energy decomposition analysis attributes this performance to cooperative electrostatic, polarization, and dispersion enhancements. Ti–B_4_N_4_ emerges as a highly promising theoretical candidate, warranting future experimental validation.

## 1. Introduction

The transition toward a carbon neutral energy system requires efficient, safe, and sustainable energy carriers capable of bridging the intermittency of renewable sources. Hydrogen has emerged as a leading candidate due to its high energy density, zero carbon combustion product, and compatibility with fuel cell technologies [1,2,3]. However, the lack of compact, reversible, and cost-effective hydrogen storage materials remains one of the key challenges hindering the large-scale deployment of the hydrogen economy [4,5]. Conventional hydrogen storage technologies, such as high pressure compression and cryogenic liquefaction, suffer from safety hazards, high energy consumption, and limited volumetric efficiency [5,6]. Consequently, solid state hydrogen storage based on adsorption and desorption processes in nanostructured materials has attracted growing attention as a safer and more energy efficient alternative [7,8,9].

In solid state systems, the ideal hydrogen adsorbent should possess high gravimetric and volumetric capacities, moderate adsorption energies (−0.10 to −0.60 eV/H_2_), and facile reversibility under near ambient conditions [10,11]. The U.S. Department of Energy (DOE) has set an ambitious target of 5.5 wt % reversible hydrogen capacity by 2025, motivating the exploration of lightweight materials composed of low atomic-weight elements such as boron, carbon, and nitrogen [12]. In this context, boron nitride (BN) based nanostructures have emerged as promising candidates owing to their high thermal stability, wide band gap, chemical inertness, and tunable electronic properties [13,14,15]. The ability of boron and nitrogen to form stable sp^2^ and sp^3^ hybridized frameworks enables the synthesis of various BN allotropes, including nanosheets, nanocages, and quantum dots, which can serve as efficient materials for molecular hydrogen adsorption [16].

In recent years, first-principles theoretical calculations have played a crucial role in advancing host-guest interaction research [17,18,19,20]. Computational modeling based on density functional theory (DFT) allows researchers to accurately predict adsorption energies, charge transfer, and thermodynamic parameters before experimental synthesis [8,21,22,23]. These calculations not only help identify promising host materials and dopants but also elucidate fundamental adsorption mechanisms, such as the nature of H_2_ surface interactions [24,25]. Furthermore, theoretical studies enable systematic screening of new materials particularly quantum confined and metal decorated systems while minimizing experimental costs and guiding the design of efficient hydrogen storage platforms. It is worth noting that nanostructured systems of comparable complexity have previously been investigated using density functional theory prior to their experimental realization. For example, studies on silicon–metal clusters have shown that DFT-predicted stable nanocage-like structures can act as building blocks for cluster-assembled materials, and were later supported by experimental observations of metal-containing silicon clusters. These findings highlight the important role of first-principles calculations as a predictive tool in guiding the synthesis and design of advanced nanomaterials [26].

Among the variety of potential nanostructures, B_4_N_4_ nanocages represent a particularly interesting subclass of boron nitride quantum dots due to their unique atomic symmetry, strong B–N covalent bonds, and pronounced quantum confinement effects. Although ultra-small BN nanocages such as B_4_N_4_ have been mainly explored through theoretical studies, their direct experimental synthesis remains challenging due to their high curvature and size limitations. Nevertheless, a wide range of boron nitride nanostructures, including nanotubes and fullerene-like cages, have been successfully synthesized and extensively characterized, demonstrating the ability of BN frameworks to stabilize curved and confined geometries [27,28]. These advances support the relevance of small BN nanocages as realistic model systems for investigating adsorption phenomena at the nanoscale.

Recent first-principles calculations by Ahmed et al. demonstrated that the pristine B_4_N_4_ nanocage exhibits a high cohesive energy (−6.23 eV), indicating excellent thermodynamic stability [29]. The optimized structure shows a balanced charge distribution between boron and nitrogen atoms, where nitrogen atoms are partially negative and boron atoms are slightly positive, reflecting the intrinsic polarity of B–N bonds. Furthermore, Ahmed et al. revealed that adsorption of a chromium atom on the B_4_N_4_ nanocage induces significant charge transfer from the metal to the cage, accompanied by a reduction in the energy gap and an increase in electronic conductivity. This behavior indicates that B_4_N_4_ nanocages can effectively stabilize metal dopants through strong metal–support interactions with partial covalent character. These findings highlight the potential of the B_4_N_4_ framework as a robust and electronically tunable platform for metal decoration, capable of preserving structural integrity while enabling controlled modulation of surface reactivity, which is essential for optimizing hydrogen adsorption and desorption processes.

In general, surface functionalization or metal decoration has been proven to be a highly effective strategy to tailor H_2_ binding energies within the optimal window required for reversible hydrogen storage [24,30,31]. In particular, alkali metals such as lithium are known to enhance hydrogen adsorption through strong charge-induced polarization effects, where the electropositive metal atom induces a dipole in the H_2_ molecule, leading to moderate and reversible interactions. This mechanism has been widely reported in both theoretical and experimental studies of carbon- and boron nitride-based nanostructures, where Li decoration improves adsorption capacity while maintaining the molecular integrity of hydrogen.

On the other hand, transition metals such as titanium introduce a fundamentally different interaction mechanism. Due to their partially filled d orbitals, Ti atoms can interact with the σ and σ* orbitals of H_2_ via the Kubas interaction, resulting in stronger yet non-dissociative binding suitable for reversible storage [31,32]. Previous studies have demonstrated that decorating BN and carbon nanostructures with light transition metals, particularly Ti, significantly enhances hydrogen uptake and storage stability [33,34]. For instance, Ti-decorated h-BN nanosheets exhibit adsorption energies in the range of −0.30 to −0.50 eV/H_2_ and reversible capacities up to 5.6 wt% [14]. Experimental and theoretical investigations have also confirmed that metal functionalization of BN-based materials is an effective approach to stabilize dispersed metal centers and improve adsorption performance.

Based on these considerations, lithium and titanium were selected in this work as representative dopants providing two complementary interaction regimes: Li as a prototype of electrostatic polarization-driven adsorption, and Ti as a model transition metal enabling stronger, partially covalent interactions through d-orbital hybridization. This dual approach allows a systematic comparison between weak-to-moderate and stronger adsorption mechanisms within the same B_4_N_4_ framework.

Despite these advances, systematic investigations of metal-decorated B_4_N_4_ nanocages or their quantum dot analogues remain scarce. The B_4_N_4_ cage offers distinctive advantages over larger BN clusters: (i) its small size and discrete structure induce quantum confinement effects that modify its frontier orbital distribution; (ii) its balanced B:N ratio ensures electronic neutrality and well-defined coordination sites for metal anchoring; and (iii) its low atomic mass can theoretically enable gravimetric hydrogen capacities well above the DOE target Furthermore, compared to extended 2D BN sheets, B_4_N_4_ quantum dots are expected to limit metal clustering due to their localized binding sites and quantum-confined geometry, which enhance metal support interactions and stabilize isolated metal atoms. Similar effects have been reported for low-dimensional carbon and BN-based nanostructures, where strong anchoring and curvature contribute to suppressing metal aggregation and maintaining high dispersion of dopant atoms [30,35]. These combined features make metal-decorated B_4_N_4_ nanocage quantum dots a highly promising platform for reversible hydrogen storage with optimized thermodynamic and kinetic performance.

In this study, we present a comprehensive density functional theory (DFT) investigation of hydrogen adsorption on metal decorated B_4_N_4_ nanocage quantum dots (M–B_4_N_4_, M = Li, Ti). Structural, electronic, and energetic properties were systematically analyzed to determine the most favorable metal dopant and hydrogen adsorption configuration. Sequential hydrogen adsorption and desorption were explored to quantify the theoretical and reversible gravimetric capacities. In addition, thermodynamic and kinetic analyses based on van’t Hoff and Arrhenius formalisms were carried out to assess the feasibility of hydrogen uptake and release under operational conditions. The findings provide new insights into the design of lightweight, metal functionalized boron nitride nanostructures for next-generation solid-state hydrogen storage applications.

## 2. Computational Details

All calculations were performed within the framework of Kohn Sham density functional theory (DFT) using the ωB97X-3c functional [36] in combination with the valence double-ζ polarized (vDZP) basis set, as implemented in the ORCA 5.0.4 quantum chemistry package [37]. The ωB97X-3c composite functional, developed by Grimme and co-workers, combines a range-separated hybrid exchange correlation formulation with a small but well-balanced basis set, an atom-pairwise D4 dispersion correction, and a geometrical counterpoise (gCP) correction for basis-set superposition error (BSSE). This combination ensures accurate geometries and reliable interaction energies for noncovalent and weakly chemisorptive systems at a modest computational cost, making it particularly suitable for the study of hydrogen adsorption on nanocage quantum dots. The pristine B_4_N_4_ nanocage, its metal-decorated forms (M–B_4_N_4_; M = Li, Ti), and the corresponding hydrogen-adsorbed complexes were fully optimized without any symmetry constraints. Self-consistent field (SCF) convergence was set to 10^−6^ a.u. for energy and 10^−8^ a.u. for density, with a maximum gradient threshold of 3 × 10^−4^ a.u. Vibrational frequency analyses were carried out at the same ωB97X-3c/vDZP level to confirm that each optimized geometry corresponds to a true minimum on the potential energy surface (no imaginary frequencies) and to obtain zero-point energy (ZPE) and finite temperature corrections at 298 K and 1 atm. For the pristine B_4_N_4_ nanocage, all frequencies were real, confirming its structural stability in agreement with earlier theoretical reports [29]. To further evaluate the intrinsic stability of the pristine and metal-decorated B_4_N_4_ nanocages, the cohesive energy (E_coh_) was also calculated. The cohesive energy per atom was obtained using the standard expression [38]:

(1) $$E_{\mathrm{coh}}=\frac{E_{\mathrm{total}}-\sum_{i}n_{i}E_{i}}{N}$$

where $E_{\mathrm{total}}$ is the total electronic energy of the fully optimized B_4_N_4_ (pristine or decorated) system, $E_{i}$ is the atomic energy of the isolated atom i, $n_{i}$ is the number of atoms of type i, and N is the total number of atoms in the nanocage. A more negative $E_{\mathrm{coh}}$ indicates a structurally more stable configuration.

The binding energy of a single Li or Ti atom on the B_4_N_4_ nanocage was calculated to quantify the strength of the metal–support interaction. For one metal atom M (M = Li or Ti) adsorbed on the nanocage, the binding energy was evaluated as

(2) $$E_{b}\left(M\right)=E_{M–B_{4}N_{4}}-E_{B_{4}N_{4}}-E_{M}$$

where $E_{M–B_{4}N_{4}}$ is the total energy of the optimized metal-decorated B_4_N_4_ nanocage, $E_{B_{4}N_{4}}$ is the total energy of the pristine B_4_N_4_ nanocage, and $E_{M}$ is the energy of the isolated Li or Ti atom in the gas phase. All energies were obtained at the same level of theory. Negative values of $E_{\mathrm{bind}}$ indicate exothermic and thermodynamically favorable binding of the metal atom to the B_4_N_4_ cage. Because ωB97X-3c inherently includes both dispersion (D4) and gCP corrections, no additional empirical or BSSE corrections were applied, ensuring internal consistency of all calculated interaction energies.

The adsorption energy for one hydrogen molecule adsorbed on a metal decorated nanocage was calculated as:

(3) $$E_{\mathrm{ads}}=E\left({H_{2}/M–B}_{4}N_{4}\right)-E\left({M–B}_{4}N_{4}\right)-E\left(H_{2}\right)\;\;$$

where $E({H_{2}/M–B}_{4}N_{4})$ is the total electronic energy of the optimized H_2_ adsorbed complex, $E\left({M–B}_{4}N_{4}\right)$ is the energy of the isolated metal-decorated nanocage, and $E(H_{2})$ is the energy of an isolated H_2_ molecule optimized at the same level of theory. Although Equations (2) and (3) share a similar mathematical form, they describe different physical interactions. Equation (2) corresponds to the binding energy of a metal atom on the B_4_N_4_ nanocage, while Equation (3) represents the adsorption energy of hydrogen molecules on the (metal-decorated) nanocage. For systems containing multiple adsorbed H_2_ molecules, the average adsorption energy per H_2_ molecule was determined as:

(4) $${\bar{E}}_{\mathrm{ads}}=\frac{1}{n}[E({M–B}_{4}N_{4}\cdot \left(H_{2}{)}_{n}\right)-E\left({M–B}_{4}N_{4}\right)-nE\left(H_{2}\right)]$$

where n  is the number of hydrogen molecules adsorbed on the metal-decorated nanocage. This expression provides an effective measure of the average interaction strength during sequential hydrogen adsorption.

The gravimetric hydrogen storage capacity (wt%) was determined using the following equation [39]:

(5) $$\mathrm{wt}\%=\frac{nM(H_{2})}{M(M–B4N4)+nM(H_{2})}\times 100$$

where $M(H_{2})$ and M(M–B4N4) are the molar masses of hydrogen and metal decorated B_4_N_4_, respectively.

The thermal stability of the pristine and metal-decorated B_4_N_4_ nanocages was evaluated through vibrational frequency analysis and cohesive energy calculations. The absence of imaginary frequencies confirms that all optimized structures correspond to true minima on the potential energy surface. In addition, the significantly negative cohesive energies indicate strong structural stability. It is worth noting that boron nitride-based nanostructures are widely recognized for their high thermal robustness over a broad temperature range, as reported in previous experimental and theoretical studies. Therefore, these results provide strong evidence for the intrinsic stability of the investigated systems.

## 3. Results and Discussion

The following section provides a comprehensive analysis of the structural stability, metal decoration behavior, and hydrogen adsorption characteristics of the B_4_N_4_ nanocage, aiming to elucidate its potential as a lightweight hydrogen storage material. Understanding the intrinsic geometry and energetics of the pristine nanocage is essential, as curvature, bond polarization, and cohesive strength dictate both its chemical reactivity and its ability to host metal dopants. The interaction of Li and Ti adatoms with B_4_N_4_ is then examined to identify the most favorable binding sites and to determine how metal decoration modifies the electronic environment of the cage, thereby activating it toward hydrogen adsorption. Subsequently, the adsorption of a single H_2_ molecule is analyzed to establish the fundamental interaction mechanism physisorption versus chemisorption and to evaluate the suitability of each metal site for reversible hydrogen binding. Finally, sequential H_2_ adsorption is investigated to assess the maximum storage capacity, adsorption energetics, and reversibility, providing key insights into the practical applicability of metal decorated B_4_N_4_ as a promising platform for solid state hydrogen storage.

### 3.1. Structural Features, Stability, and Metal Binding Behavior of the B_4_N_4_ Nanocage

**Figure 1:**
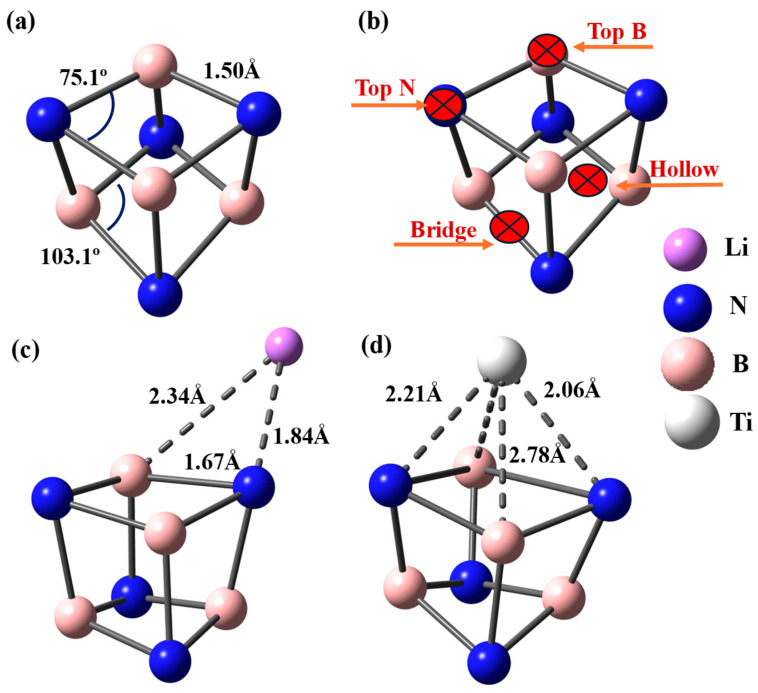
(**a**) Optimized atomic structure of B_4_N_4_ quantum dot using ωB97X-3c/vDZP level of theory and (**b**) their possible adsorption sites. (**c**,**d**) represent the most stable optimized geometries of Li-B_4_N_4_ and Ti-B_4_N_4_, respectively.

The pristine B_4_N_4_ quantum dot displays a highly curved and compact cage-like structure, as illustrated in Figure 1a, with a uniform B–N bond length of 1.50 Å and significantly distorted angular environments (B–N–B = 75.10° and N–B–N = 103.10°). These structural features arise from the geometric confinement of the eight-membered BN ring and reflect the intrinsic curvature-induced strain characteristic of nanoscale BN clusters. The optimized geometry is in excellent agreement with the theoretical findings of Ahmed et al., who reported a similar B–N distance of 1.504 Å for a comparable B_4_N_4_ quantum dot, confirming the robustness and reproducibility of this structural motif across different DFT methodologies. Such agreement supports the reliability of the ωB97X-3c/vDZP level of theory used in this work, particularly for systems combining curvature, polarization, and weakly covalent bonding.

The electronic distribution within the pristine nanocage further corroborates its polar and asymmetric nature. Mulliken charge analysis (Table S1) reveals that nitrogen atoms bear negative charges of approximately −0.235 e, while boron atoms carry positive charges of about +0.235 e, reflecting the well-known electronegativity imbalance of BN networks. This charge separation gives rise to distinct adsorption environments, which are schematically illustrated in Figure 1b: (i) the electron-rich Top N site, (ii) the electrophilic Top B site, (iii) the intermediate Bridge site located above a B–N bond, and (iv) the nearly symmetric Hollow site centered above the cage cavity. These four sites constitute the fundamental interaction landscape of the quantum dot and define how external species such as metal atoms or hydrogen molecules may bind to the surface.

Upon metal decoration, the B_4_N_4_ cage exhibits different structural responses depending on whether Li or Ti is adsorbed. In the case of Li, the metal prefers a bridge configuration slightly shifted toward a nitrogen atom, as shown in Figure 1c, leading to an asymmetric coordination with Li–N = 1.84 Å and Li–B distances of 1.67 and 2.34 Å. This binding geometry reflects the strong electrostatic attraction between the electropositive Li atom and the negatively charged N atoms. The local BN environment undergoes only minor distortions: B–N bonds near the adsorption site expand modestly (up to 1.67 Å), and the angles adjust slightly to B–N–B = 74.34° and N–B–N = 97.48° (Table 1). Importantly, the global curvature of the nanocage is largely preserved, and the cohesive energy remains significantly negative (−5.75 eV), indicating that Li decoration does not disrupt the cage stability. The predicted binding energy (−2.30 eV) combined with the moderate metal charge (+0.51 e, Table S1) suggests that Li interacts predominantly through ionic and electrostatic pathways, creating moderately active adsorption sites for hydrogen.

In contrast, Ti adsorption induces a structural reconstruction of the nanocage, as seen in Figure 1d. The Ti atom does not bind directly atop a single atom but instead occupies a distorted hollow position, slightly displaced toward a center of the N–B–N triangle. This results in multi-center interactions involving Ti–N distances of 2.21 and 2.06 Å and Ti–B distances of 2.06 and 2.37 Å, forming a more delocalized coordination than in the Li case. The impact on the BN framework is far more pronounced: B–N bonds around Ti are significantly stretched up to 2.78 Å and the angular environment is heavily reorganized, with B–N–B expanding to 88.54° while N–B–N collapses to 54.30° (Table 1). These local deformations indicate that Ti forms a partially covalent interaction with the nanocage, consistent with its larger positive charge (+0.82 e) and its well-known tendency to hybridize d-orbitals with surrounding p-orbitals. Despite these stronger structural perturbations, the Ti-decorated nanocage remains energetically stable, with a cohesive energy of −6.07 eV close to that of the pristine system and a high binding energy (−5.24 eV).

To further understand the impact of Ti adsorption on the nanocage, it is instructive to compare the Ti–B and Ti–N interactions with the intrinsic B–N bonding of the pristine structure. In the undoped B_4_N_4_ nanocage, the B–N bonds are short (~1.50 Å) and exhibit strong covalent character. Upon Ti adsorption, these bonds are significantly elongated (up to ~2.78 Å in the vicinity of the metal center), indicating a local weakening of the B–N framework due to charge redistribution and orbital hybridization with Ti.

In contrast, the Ti–B and Ti–N distances (2.06–2.37 Å and 2.06–2.21 Å, respectively) reflect the formation of stable metal–support interactions with partial covalent character. The high binding energy of Ti (−5.24 eV) further confirms the strength of these interactions. Overall, the formation of strong Ti–B and Ti–N bonds offsets for the local distortion of the B–N network, preserving the global structural stability of the nanocage.

The combined structural and electronic results summarized in Table 1 and Table S1 demonstrate that Li and Ti produce two distinct interaction regimes on B_4_N_4_: Li forms moderately strong, predominantly ionic bonds that perturb the nanocage only locally, while Ti establishes strongly bound, highly polarized adsorption centers that induce deeper rearrangements of the BN network. Importantly, both systems maintain overall structural integrity and produce well-defined metal sites that are expected to play a crucial role in governing hydrogen uptake and release. These findings provide a solid foundation for the analysis of H_2_ adsorption mechanisms presented in the following sections.

### 3.2. Single H_2_ Adsorption Mechanism on B_4_N_4_, Li–B_4_N_4_, and Ti–B_4_N_4_

Building on the structural and electronic insights obtained in the previous section, the adsorption behavior of a single hydrogen molecule on the pristine and metal-decorated B_4_N_4_ nanocages can now be examined in detail. The distinct structural and electronic characteristics of pristine and decorated nanocage are expected to play a decisive role in determining how the first H_2_ molecule interacts with each system. Understanding the mechanism of this initial adsorption step is essential, as it dictates the nature of the binding interaction, the degree of H–H activation, and the suitability of each metal site for subsequent hydrogen uptake. Therefore, the following section examines the geometrical modifications, binding energies, and electronic signatures associated with the adsorption of a single H_2_ molecule on pristine B_4_N_4_, Li–B_4_N_4_, and Ti–B_4_N_4_, providing the foundation for assessing their hydrogen storage potential.

**Figure 2:**
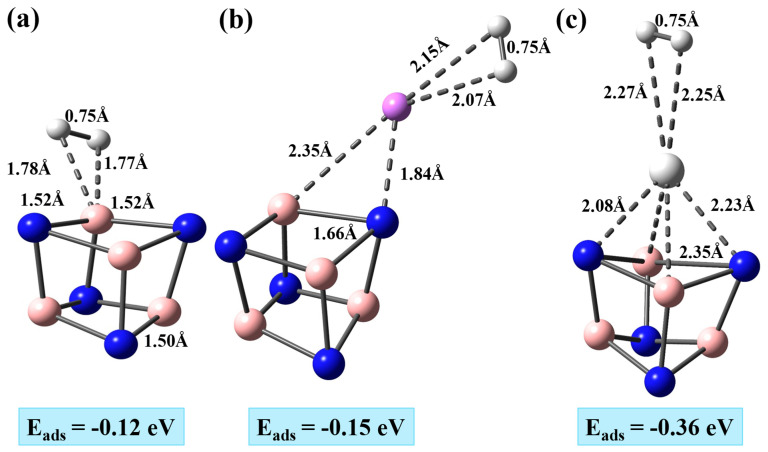
Most stable configurations of H_2_ adsorption on (**a**) pristine B_4_N_4_, (**b**) Li-decorated B_4_N_4_, and (**c**) Ti-decorated B_4_N_4_ nanocages, along with the corresponding adsorption distances and energies. White, blue, and pink spheres represent hydrogen, nitrogen, and boron atoms, respectively.

As in the case of metal adsorption, four distinct adsorption sites pristine B_4_N_4_ were considered: the Top N, the Top B, the Bridge and the Hollow sites. For each site, two different orientations (parallel and perpendicular) of the H_2_ molecule were considered in order to capture the possible binding configurations driven by electrostatic interactions and local anisotropy of the electronic density. In the parallel orientation, the H_2_ molecular axis lies approximately tangent to the cage surface, allowing both hydrogen atoms to interact symmetrically with the adsorption site. In the perpendicular orientation, the H_2_ axis is oriented normal to the cage surface, such that one hydrogen atom points toward the adsorption center while the second hydrogen points away from the surface. The inclusion of these two orientations is essential, as BN-based materials often show orientation-dependent adsorption due to the strong heteropolar character of the B–N bonds. After the geometry’s optimization, the most stable configuration of a single H_2_ molecule corresponds to adsorption at the Top B site in a parallel orientation relative to the cage surface (Figure 2a). In this geometry, the shortest H–B distance is 1.77 Å, while the H–H bond length is slightly elongated to 0.75 Å compared to 0.74 Å in the isolated gas-phase molecule. The very small increase in the H–H distance indicates that the dihydrogen molecule remains essentially intact and only weakly perturbed upon adsorption, which is consistent with a predominantly physisorptive interaction. The B–N bond nearest to the adsorption site is marginally stretched from 1.50 Å in the pristine cage to 1.52 Å in the H_2_–B_4_N_4_ complex, confirming that the interaction does not induce significant structural distortion in the framework. The calculated adsorption energy, $E_{\mathrm{ads}}=-0.12$ eV, further supports this picture of weak binding. Overall, these results demonstrate that the pristine B_4_N_4_ nanocage provides only a weakly interacting surface for H_2_, and therefore mainly serves as a reference to quantify the enhancement in binding strength and activation that arises upon Li and Ti decoration in the following subsections.

The weak adsorption energy obtained for a single H_2_ molecule on pristine B_4_N_4_ (−0.12 eV) is fully consistent with the general behavior of boron nitride-based nanomaterials. Numerous first-principles studies have shown that pristine BN sheets and BN nanotubes interact only weakly with molecular hydrogen, with adsorption energies typically between −0.05 and −0.15 eV, reflecting purely physisorptive interactions governed by van der Waals forces rather than charge transfer or orbital hybridization [40,41]. These works also consistently report that H_2_ remains almost unperturbed upon adsorption, with H–H elongations smaller than 0.02–0.03 Å, in line with the very small increase observed here (0.74 → 0.75 Å) [40].

A first enhancement in adsorption strength is observed upon Li decoration. The most stable configuration places the H_2_ molecule in a parallel orientation above the Li atom (Top Li site), with Li–H distances of 2.07 and 2.15 Å, and a slight elongation of the H–H bond to 0.75 Å (Figure 2b). The Li–N and Li–B distances are 1.81 and 2.35 Å, respectively, while the adjacent B–N bond elongates to 1.66 Å, indicating modest local deformation of the cage. The adsorption energy of −0.15 eV shows that Li enhances the interaction compared to the pristine surface, in agreement with the expected charge induced dipole mechanism typical of alkali-metal decoration. When compared to Li-decorated B_4_N_4_ sheets reported in the literature, which show adsorption energies between −0.18 and −0.22 eV for the first H_2_ molecule [42], the nanocage presents a slightly lower binding energy but shorter Li–H distances (2.07–2.15 Å vs. ≈2.11–2.17 Å on the sheet). This difference originates from the curvature of the nanocage, which confines the local electrostatic field more strongly than the planar sheet and allows H_2_ to approach Li more closely. The nanocage also undergoes a larger local deformation (B–N stretched to 1.66 Å) than the 2D sheet, reflecting the greater structural flexibility of the curved geometry of B_4_N_4_ quantum dot.

The Ti-decorated nanocage shows a markedly stronger interaction with H_2_. The most stable configuration corresponds to a parallel orientation above the Ti atom (Top Ti site), where Ti–H distances of 2.25 and 2.27 Å are accompanied by an adsorption energy of −0.36 eV, more than double that observed for Li and almost triple that of the pristine cage (Figure 2c). The BN framework undergoes significant reconstruction, with Ti–N and Ti–B distances of 2.08 and 2.23 Å and a pronounced elongation of the nearest B–N bond to 2.75 Å. While the H–H bond remains essentially molecular at 0.75 Å, the stronger interaction reflects partial hybridization between Ti 3d orbitals and the σ/σ* orbitals of H_2_, producing a weak Kubas-type contribution [31]. This deeper electronic coupling explains both the higher adsorption energy and the greater structural deformation observed for Ti–B_4_N_4_.

These results clearly show that metal decoration substantially enhances hydrogen adsorption on the B_4_N_4_ nanocage, with transition-metal doping producing the largest effect. Li provides a moderately polar adsorption center capable of slightly activating H_2_ while preserving its molecular character, whereas Ti generates a significantly stronger interaction through combined polarization and orbital hybridization. According to Figure 2, the adsorption energies follow the order $\mathrm{Ti}–B_{4}N_{4}>\mathrm{Li}–B_{4}N_{4}>\mathrm{Pristine}\;B_{4}N_{4},$ demonstrating the strong enhancement produced by metal dopants and motivating the exploration of sequential hydrogen adsorption.

To further clarify the relationship between electronic structure and single-molecule adsorption behavior, a frontier molecular orbital (FMO) analysis was performed for pristine and metal-decorated B_4_N_4_ nanocages before and after adsorption of one H_2_ molecule. The results (Table S2, Supporting Information) show that the pristine B_4_N_4_ nanocage possesses a large HOMO–LUMO gap (9.90 eV), which remains essentially unchanged after H_2_ adsorption (9.89 eV), confirming the weak physisorptive interaction discussed above.

In contrast, metal decoration significantly reduces the HOMO–LUMO gap to 6.42 eV for Li–B_4_N_4_ and 7.86 eV for Ti–B_4_N_4_, indicating enhanced electronic reactivity induced by charge redistribution and metal–support interactions. However, after adsorption of a single H_2_ molecule, only very small changes are observed in the HOMO, LUMO, and ΔEg values. This indicates that although metal decoration modifies the intrinsic electronic structure of the nanocage and activates it toward adsorption, the actual H_2_ binding is mainly governed by localized interactions at the metal center rather than by large global changes in the electronic structure. These results are fully consistent with the proposed single-molecule adsorption mechanisms, where Li enhances adsorption through charge-induced polarization, while Ti promotes stronger binding through d-orbital hybridization.

### 3.3. Mechanistic Insights into Sequential H_2_ Adsorption and Reversible Storage on Pristine and Metal-Decorated B_4_N_4_ Quantum Dots

To further assess the hydrogen storage performance of pristine and metal-decorated B_4_N_4_ quantum dots, it is essential to examine their behavior under sequential hydrogen loading. While single molecule adsorption provides fundamental insight into the intrinsic interaction strength of each adsorption site, practical hydrogen storage requires evaluating how the system responds as multiple H_2_ molecules are progressively added.

**Figure 3:**
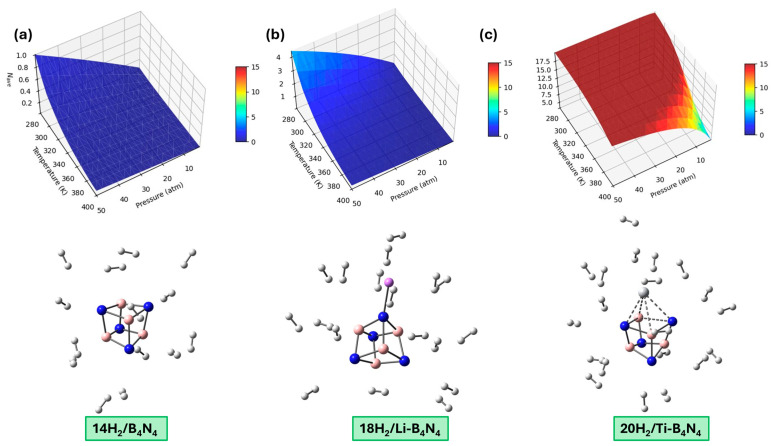
Average number of adsorbed H_2_ molecules of hydrogen and optimized saturated geometries of (**a**) pristine B_4_N_4_ (**b**) Li-B_4_N_4_ and (**c**) Ti-B_4_N_4_. White, blue, pink, purple, and light gray spheres represent hydrogen, nitrogen, boron, lithium, and titanium atoms, respectively.

For the pristine B_4_N_4_ nanocage, sequential adsorption was investigated up to the maximum loading capacity of 14 hydrogen molecules (Figure 3a), which corresponds to the complete saturation of all accessible adsorption sites. The average adsorption energy for a configuration containing n hydrogen molecules, denoted ${\bar{E}}_{ads}$, was calculated using Equation (4), which provides an effective measure of the interaction strength as the number of adsorbed molecules increases. ${\bar{E}}_{ads}$ decreases steadily from −0.12 eV/H_2_ for the first molecule to approximately −0.06 eV/H_2_ at saturation (14 H_2_). This monotonic weakening reflects the physisorptive nature of the interaction, dominated by weak van der Waals forces that remain nearly constant for isolated molecules but become increasingly screened as the number of adsorbed H_2_ grows. No significant structural changes are induced in the nanocage, and the H–H bonds remain essentially unperturbed, indicating that pristine B_4_N_4_ is unable to promote cooperative or enhanced adsorption. The progressive reduction in interaction strength demonstrates that hydrogen adsorption on the undoped nanocage is purely physical and becomes marginal when multiple H_2_ molecules accumulate, which limits its relevance for practical storage applications.

For the Li-decorated nanocage, sequential adsorption exhibits a different trend. The first adsorbed H_2_ molecule interacts with an average adsorption energy of −0.15 eV/H_2_, reflecting the polarization induced by the electropositive Li atom. As additional molecules are introduced, ${\bar{E}}_{ads}$ decreases gradually but remains within the desirable reversible-storage window (−0.10 to −0.15 eV/H_2_) up to approximately three hydrogen molecules. Beyond this point, the Li-centered electric field becomes progressively screened, and ${\bar{E}}_{ads}$ drops toward values typical of weak physisorption (≈−0.06 to −0.08 eV/H_2_). At saturation, the Li–B_4_N_4_ nanocage accommodates up to 18 H_2_ molecules (Figure 3b) due to the strong curvature of the quantum dot, which creates multiple dispersion sites around the cage. Despite this high gravimetric capacity, the adsorption remains largely non-cooperative: the interaction weakens smoothly as each additional H_2_ molecule is added, indicating that Li provides only a limited activation of the nanocage. The overall adsorption behavior is therefore dominated by initial polarization effects followed by van der Waals interactions at higher loadings.

The Ti-decorated nanocage shows the most distinctive sequential adsorption behavior among the three systems. The first hydrogen molecule binds strongly with an average adsorption energy of −0.36 eV/H_2_, reflecting the partially covalent and polarization enhanced interaction facilitated by the Ti center. As more H_2_ molecules are adsorbed, ${\bar{E}}_{ads}$ decreases but remains significantly higher than in the Li-decorated and pristine cases for a wide range of loadings (up to 8–9 H_2_). This indicates that the electronic environment around Ti enables cooperative stabilization, likely through multi-center polarization and weak Kubas-type interactions involving Ti d orbitals. Even at higher coverages, where crowding effects reduce the available interaction space, the average adsorption energy remains within a moderate and thermodynamically meaningful range (−0.10 to −0.14 eV/H_2_). The Ti–B_4_N_4_ nanocage can accommodate up to 20 H_2_ molecules (Figure 3c), and unlike the pristine or Li-decorated systems, the decrease in ${\bar{E}}_{ads}$ does not follow a simple monotonic physisorption trend. Instead, the Ti center continues to influence the surrounding adsorption environment, maintaining partially activated interactions even at elevated loadings. This behavior highlights the cooperative and electronically mediated nature of sequential adsorption on Ti-modified B_4_N_4_, which is essential for achieving practical hydrogen storage under mild conditions.

To evaluate the reversibility of hydrogen storage on pristine and metal-decorated B_4_N_4_ quantum dots, the desorption behavior of sequentially adsorbed H_2_ molecules was examined using three complementary parameters: the desorption energy $E_{des}$, the desorption temperature $T_{D}$, and the desorption time τ. These parameters, summarized in Table 2 and Table 3, provide a complete description of the thermodynamic and kinetic feasibility of releasing hydrogen under practical conditions.

The desorption energy for the n-th hydrogen molecule was evaluated using the incremental expression

(6) $$E_{des}(n)=E_{nH_{2}/{M–B}_{4}N_{4}}-[E_{(n-1)H_{2}/{M–B}_{4}N_{4}}+E_{H_{2}}]$$

which quantifies the energy required to remove the last adsorbed H_2_ molecule from the complex. Because this definition directly mirrors the adsorption process, the magnitude of $E_{des}$ provides a clear and physically intuitive indication of hydrogen retention: a smaller $E_{des}$ value implies weaker binding and therefore easier desorption, fully consistent with the evolution of the average adsorption energy ${\bar{E}}_{ads}$ discussed earlier.

For the pristine B_4_N_4_ nanocage, the values of $E_{des}$ listed in Table 2 remain very small (≈0.05–0.08 eV) across all loadings, matching the weak van der Waals adsorption energies. The small magnitude of $E_{des}$ confirms that each H_2_ molecule is only loosely retained and can be desorbed with minimal energetic cost, which is in full agreement with the monotonic decrease of ${\bar{E}}_{ads}$ from −0.12 to −0.06 eV/H_2_ as saturation is approached.

For the Li-decorated nanocage, the first few hydrogen molecules exhibit moderately larger $E_{des}$ values (≈0.12–0.14 eV), reflecting the polarization-enhanced adsorption induced by Li. As the number of adsorbed molecules increases, $E_{des}$ rapidly decreases to the physisorption range (≈0.05–0.06 eV), in parallel with the reduction of ${\bar{E}}_{ads}$. This simultaneous decline in both adsorption and desorption energies indicates that once the Li electric field becomes screened, additional hydrogen molecules are only weakly stabilized and are therefore more easily released.

The Ti-decorated nanocage shows the strongest incremental desorption energies, especially for the first adsorbed molecules, where $E_{des}$ reaches values up to −0.35 eV. This is fully consistent with the higher initial ${\bar{E}}_{ads}$ (−0.36 eV/H_2_) driven by the polarized and partially Kubas-like Ti–H_2_ interaction. As the loading increases, Ti retains relatively large $E_{des}$ values compared to Li and pristine systems, confirming its superior ability to stabilize multiple H_2_ molecules. Only at high coverage does $E_{des}$ decrease into the weaker physisorption regime, matching the gradual drop in ${\bar{E}}_{ads}$ observed near saturation.

After establishing the sequential adsorption trend, the practical reversibility of hydrogen storage was evaluated also through the desorption temperature $T_{D}$ and the desorption time τ, which provide thermodynamic and kinetic indicators of H_2_ release under operating conditions. In this work, the desorption temperature was estimated using the Van’t Hoff equation [43]:

(7) $$T_{D}=-\frac{E_{\mathrm{ads}}}{k_{B}\;(\frac{\Delta S}{R}-lnP)}$$

T_D_ incorporates variables such as adsorption energy (E_ads_), the equilibrium pressure (P), and the entropy change (ΔS = 75.44 J mol^−1^ K^−1^) of hydrogen molecules transitioning from gaseous phase to liquid phase. Additionally, k_B_ and R represent the Boltzmann constant and the ideal gas constant, respectively. This expression predicts higher desorption temperatures at higher pressure because the driving force for desorption decreases when P increases. The kinetic desorption time was evaluated through an Arrhenius/transition-state- equation [44]:

(8) $$\tau =\frac{exp(-E_{\mathrm{ads}}/k_{B}T)}{\nu_{0}}$$

where *v*_0_ is the attempt frequency (10^12^ s^−1^).

The values in Table 3 show that pristine B_4_N_4_ exhibits very low desorption temperatures, $T_{D}=115.1\;K$ at 1 bar and only 184.1 K at 30 bar, rising modestly to 233.6 K at 100 bar. This confirms that hydrogen is thermodynamically unstable on pristine B_4_N_4_ above cryogenic conditions, consistent with its weak physisorption regime. The corresponding desorption times are extremely short, decreasing from 0.21 ns at 233 K to 0.06 ns at 298 K and 0.02 ns at 400 K. Such ultrafast kinetics signify that once temperature approaches ambient conditions, H_2_ desorbs almost instantaneously, leaving negligible reversible storage despite the theoretical capacity.

Li–B_4_N_4_ yields slightly improved desorption thermodynamics. The desorption temperature increases to 140.6 K at 1 bar and 224.9 K at 30 bar, reaching 285.6 K only at 100 bar. This indicates that Li decoration stabilizes H_2_ more than the pristine cage but still requires elevated pressures to approach near-ambient retention. Kinetically, Li–B_4_N_4_ also remains in the rapid-release regime: τ decreases from 0.89 ns (233 K) to 0.18 ns (298 K) and 0.04 ns (400 K). Therefore, Li enables modest stabilization of the first few adsorbed molecules, but most hydrogen remains weakly bound and desorbs quickly at room temperature.

In contrast, Ti–B_4_N_4_ shows a clearly superior reversible-storage signature. The desorption temperature reaches 287.7 K already at 1 bar and increases strongly with pressure to 400.1 K at 30 bar and 484.1 K at 100 bar. These values demonstrate that Ti decoration allows hydrogen to remain thermodynamically stable under near-ambient conditions, in line with its stronger polarized/Kubas-like adsorption regime identified in Section 3.1 and Section 3.2. The desorption times further confirm this enhanced retention: Ti–B_4_N_4_ displays τ=0.30 ms at 233 K and remains orders of magnitude larger than the other systems at ambient temperature (τ=0.61 μs at 298 K, decreasing to 0.02 μs only at 400 K). Such intermediate microsecond-scale kinetics are characteristic of practical reversible storage, where H_2_ is stabilized long enough to be retained but can still be released without excessive thermal penalty.

Overall, the pressure-dependent $T_{D}$ and temperature-dependent τ trends consistently support the adsorption-energy hierarchy established earlier. Pristine B_4_N_4_ is only suitable for cryogenic physisorption, Li–B_4_N_4_ provides limited stabilization with fast desorption, whereas Ti–B_4_N_4_ achieves near-ambient thermodynamic stability and substantially slower release kinetics, confirming its strongest potential for reversible hydrogen storage.

While the saturation capacities reported above correspond to the idealized adsorption limit at 0 K, they do not account for the essential thermodynamic contributions of finite pressure and temperature. In realistic operating conditions, hydrogen adsorption and desorption occur under non-zero T and P, which modify the effective chemical potential of hydrogen and determine the real number of molecules that can be stored or released. Therefore, a thermodynamic analysis was performed to evaluate the practical absorption–desorption behavior of H_2_ on pristine and metal-decorated B_4_N_4_ nanocages.

According to the grand canonical formalism described by Equations (9) and (10), the realistic hydrogen storage performance of pristine and metal-decorated B_4_N_4_ quantum dots can be evaluated by computing the average number of adsorbed molecules $N_{\mathrm{avg}}(P,T)\;$ under practical operating conditions. In this framework, the adsorption probability of each configuration enters through the grand canonical partition function (*z*). The equation to calculate (*z*) is defined as follows [45]:

(9) $$z=1+\sum_{i=1}^{n}exp(\frac{E_{i,\mathrm{ads}}-\mu (P,T)}{k_{B}T})$$

where *n* is the maximum number of adsorbed H_2_ molecules, $E_{i,\mathrm{ads}}$ is the adsorption energy of the *i*th H_2_ molecule, $k_{B}$ is the Boltzmann constant (1.38 × 10^−23^ J K^−1^), and μ(P,T) is the chemical potential of molecular hydrogen in the gas phase at a given pressure (*P*) and temperature (*T*).

The chemical potential μ(P,T) can be determined from thermodynamic relations or from experimental data, and it is given by:

(10) $$\mu_{H_{2}}(P,T)=\Delta H+T\Delta S+k_{B}Tln\frac{P}{P_{0}}$$

where ΔH, ΔS, and $P_{0}$ represent the enthalpy change, entropy change, and standard atmospheric pressure (1.01 × 10^5^ Pa), respectively. In this expression, the term ΔH+TΔS describes the free energy contribution of the hydrogen gas phase, while the logarithmic term accounts for the dependence on external pressure. The values of ΔH and ΔS were taken from the experimental database [46], which provides reliable thermodynamic parameters for H_2_ adsorption and desorption on metal-decorated systems.

The average number of adsorbed hydrogen molecules at a given pressure and temperature, $N_{\mathrm{avg}}(P,T)$, is derived from the partition function and is expressed as:

(11) $$N_{\mathrm{avg}}(P,T)=N_{T}[\frac{z-1}{z}]$$

where $N_{T}$ denotes the number of adsorbed H_2_ molecules at 0 K (corresponding to the maximum adsorption capacity determined from DFT. Table 3 and Figure 3 display the resulting values of $N_{A}$ (number of hydrogen molecules adsorbed at charging conditions: 30 bar, 298 K), $N_{D}$ (number retained at discharging conditions: 3 bar, 373 K), and the reversible capacity $N_{P}=N_{A}-N_{D}$. For pristine B_4_N_4_, despite its theoretical saturation limit of 14 H_2_ (22.1 wt%), the calculated adsorption under realistic charging conditions is extremely small ($N_{A}=0.37$), which is clearly reflected in the almost flat profile observed in Figure 3a. This poor performance originates from the weak physisorption energies of pristine B_4_N_4_ (Table 2), which cannot compete with the relatively large thermal and entropic contributions embedded in μ(P,T). Consequently, the partition function remains close to unity and the probability of retaining hydrogen at 298 K is nearly zero. As expected, desorption is complete under discharging conditions ($N_{D}\approx 0$), confirming that pristine B_4_N_4_ cannot operate as a practical hydrogen storage material at ambient temperature.

For Li-decorated B_4_N_4_, Table 3 indicates a modest improvement. The system retains $N_{A}=1.73$ H_2_ molecules at 30 bar and 298 K, consistent with the slightly enhanced interaction energies of the first adsorbed hydrogens. Figure 3b also shows a small but noticeable increase compared to the pristine nanocage. However, because Li-induced polarization becomes quickly screened as loading increases, the incremental adsorption energies drop rapidly, which reduces the corresponding Boltzmann factors in Equation (9). As a result, only a small fraction of hydrogen remains adsorbed at ambient conditions, and nearly all molecules desorb at 3 bar and 373 K ($N_{D}=0.03$). The resulting reversible capacity ($N_{P}=1.70$, corresponding to 3.1 wt%) remains well below the practical target for onboard hydrogen storage.

By contrast, Ti-decorated B_4_N_4_ exhibits a markedly superior adsorption profile under the same charging conditions. Table 3 shows that Ti–B_4_N_4_ retains almost its entire theoretical capacity ($N_{A}=19.89$, out of a maximum of 20 H_2_), demonstrating that the strong sequential adsorption energies (Table 2) significantly outweigh the entropic penalty at 298 K and 30 bar. This behavior is clearly illustrated in Figure 3c, where the adsorption curve remains high throughout the entire loading range. Upon switching to discharging conditions (3 bar, 373 K), the average number of adsorbed molecules decreases to $N_{D}=6.80$, indicating that a substantial fraction of hydrogen is released spontaneously. The difference between charging and discharging, $N_{P}=13.20$, corresponds to an effective reversible storage capacity of 15.1 wt%, the highest among all studied systems. This result demonstrates that Ti decoration provides a unique balance between sufficiently strong adsorption to ensure uptake at ambient conditions and sufficiently moderate binding energies to permit release at elevated temperature and lower pressure.

Overall, the grand canonical thermodynamic analysis of Table 3 and Figure 3 reveals a clear performance hierarchy: pristine B_4_N_4_ retains virtually no hydrogen at 298 K, Li–B_4_N_4_ exhibits limited practical adsorption, while Ti–B_4_N_4_ achieves both high uptake at charging conditions and significant release at discharging conditions. These findings confirm that Ti-functionalized B_4_N_4_ quantum dots possess the most favorable near-ambient thermodynamic profile for reversible hydrogen storage, in full consistency with the sequential adsorption and desorption energetics presented earlier.

To better contextualize the hydrogen storage performance of the B_4_N_4_ nanocage, a comparative overview of representative lightweight nanostructured materials is presented in Table 4. As shown, conventional carbon-based materials such as graphene, carbon nanotubes, and fullerene derivatives typically exhibit moderate hydrogen storage capacities in the range of ~4–10 wt%, depending on the nature of the dopant and structural defects [11,30,47]. Similarly, defect-engineered graphene and biphenylene-based systems can achieve slightly improved capacities due to enhanced adsorption sites and metal anchoring effects [45,48].

Porous frameworks such as MOFs and COFs generally provide hydrogen storage capacities below 10 wt%, although their performance can be enhanced through appropriate metal functionalization [50,51,52,53,54,55,56,57]. These materials benefit from high surface area and tunable porosity, but their relatively large mass often limits the achievable gravimetric capacity.

In contrast, more advanced low-dimensional systems, particularly quantum-confined nanostructures and metal-engineered 2D materials, demonstrate significantly higher storage capacities. Notably, irida-graphene and quantum dot systems such as C_8_ and C_3_B_2_ exhibit exceptional performances exceeding 20 wt%, highlighting the crucial role of strong metal–support interactions and optimized adsorption energetics [49,58,59].

Within this context, the B_4_N_4_ nanocage shows a clear enhancement in hydrogen storage upon metal decoration. While the pristine structure exhibits a low capacity (0.8 wt%), Li functionalization leads to a moderate improvement (3.1 wt%), consistent with polarization-driven adsorption. More importantly, Ti decoration results in a substantial increase to 15.1 wt%, positioning the B_4_N_4_ nanocage among the high-performing materials and outperforming many conventional nanostructured systems. It should be noted that the reported values are obtained under different computational or experimental conditions; therefore, the comparison provides a general perspective on relative performance rather than a strict quantitative ranking.

While the sequential adsorption and thermodynamic analyses presented above clarify the macroscopic trends governing hydrogen uptake and release on pristine and metal-decorated B_4_N_4_ nanocages, they do not explicitly reveal the underlying physical forces responsible for these behaviors. In particular, the markedly stronger and more reversible adsorption observed for Ti–B_4_N_4_ suggests the involvement of specific interaction components beyond simple van der Waals stabilization. To elucidate the microscopic origins of these differences and quantify the relative contributions of electrostatic, polarization, dispersion, and repulsive forces, an energy decomposition analysis was carried out for both single and multiple H_2_ adsorption configurations.

### 3.4. Mechanistic Insight into Single and Multiple H_2_ Adsorption via Energy Decomposition Analysis

To gain deeper mechanistic insight into the nature of H_2_ binding on pristine and metal-decorated B_4_N_4_ nanocages, we performed a localized molecular orbital energy decomposition analysis (LMOEDA) following the scheme developed by Su and Li [60]. All energy partitioning calculations were carried out using the GAMESS-US package [61,62]. Because LMOEDA is not implemented for the ωB97X-3c functional employed in the main DFT calculations, the M06-2X/def2-TZVP level of theory was adopted for the decomposition analysis, as it is widely recognized for providing reliable descriptions of polarization, electrostatic, and dispersion contributions in weakly bound systems.

This methodology allows the total interaction energy between the adsorbent and hydrogen molecules to be separated into its principal physical components: electrostatic, exchange, polarization, dispersion, and repulsion, thereby enabling a detailed assessment of the factors governing adsorption strength. The analysis was conducted for both single-molecule adsorption (1H_2_/B_4_N_4_, 1H_2_/Li–B_4_N_4_, 1H_2_/Ti–B_4_N_4_) and fully saturated complexes (14H_2_/B_4_N_4_, 18H_2_/Li–B_4_N_4_, 20H_2_/Ti–B_4_N_4_), allowing direct comparison of interaction mechanisms across different loading regimes. The numerical values of each energy component are summarized in Table 5.

The energy decomposition analysis highlights the key role of attractive energies, such as electrostatic, polarization, and dispersion interactions, in driving the hydrogen adsorption capacity of B_4_N_4_-based materials.

The LMOEDA results provide a detailed picture of the physical forces governing hydrogen adsorption on pristine and metal-decorated B_4_N_4_ nanocages. For pristine B_4_N_4_, the interaction with a single H_2_ molecule is dominated by moderate electrostatic (−0.62 eV) and polarization (−0.96 eV) contributions, partially counterbalanced by repulsion. Although dispersion also plays a non-negligible role (−0.43 eV), the overall interaction remains weak (−0.13 eV), consistent with the physisorptive nature and the minimal activation of H_2_ observed in previous sections. In the fully saturated 14H_2_/B_4_N_4_ complex, all attractive components scale cumulatively with the number of hydrogen molecules, but the stabilizing terms remain insufficient to overcome the strong repulsion generated at high coverage. This confirms that pristine B_4_N_4_ interacts mainly through weak van der Waals forces, which explains its negligible reversible capacity at ambient conditions.

Li decoration alters the interaction landscape, but only moderately. The single-H_2_ complex with Li–B_4_N_4_ exhibits slightly reduced electrostatic (−0.10 eV) and polarization (−0.08 eV) contributions compared to pristine B_4_N_4_, while dispersion remains relatively weak (−0.08 eV). Although repulsion is significantly lower due to Li-induced charge redistribution, the total interaction (−0.11 eV) remains in the physisorption regime. At full loading (18H_2_/Li−B_4_N_4_), electrostatic and dispersion components increase as expected from cumulative molecular interactions; however, their magnitude remains modest, and repulsion rises proportionally. This energetic profile corroborates the sequential adsorption results, where Li stabilizes only the first few hydrogen molecules. Beyond this regime, the interaction becomes essentially dispersion-driven and insufficient to ensure significant reversible storage.

In contrast, Ti functionalization induces a fundamentally different adsorption mechanism. The single-H_2_/Ti–B_4_N_4_ complex exhibits markedly stronger electrostatic (−0.47 eV), polarization (−1.03 eV), and dispersion (−0.37 eV) contributions relative to the pristine and Li-decorated systems. This enhancement reflects the ability of Ti to strongly polarize H_2_ and partially hybridize with its σ orbitals, resulting in a more robust yet reversible interaction. Although repulsion is also larger due to the presence of the metal center, the sum of attractive terms dominates, yielding the most favorable total interaction energy among the three systems.

At saturation (20H_2_/Ti–B_4_N_4_), the cooperative strengthening of electrostatic, polarization, and dispersion components becomes even more pronounced. These attractive terms increase significantly as additional H_2_ molecules interact with the highly polarizable Ti center and its induced field. While repulsion grows at high loading, the combined attractive contributions (electrostatic: −1.96 eV, polarization: −2.12 eV, dispersion: −2.71 eV) are sufficient to maintain a strongly stabilizing net interaction (−2.24 eV). This cooperative energetic behavior directly supports the superior sequential adsorption capacity observed for Ti–B_4_N_4_ and explains why the system retains nearly its full theoretical hydrogen loading under ambient charging conditions.

Overall, the energy decomposition analysis reveals that Ti decoration fundamentally reshapes the interaction landscape by simultaneously enhancing multiple attractive components, rather than relying on a single dominant mechanism. This multicomponent stabilization, absent in pristine and Li-decorated nanocages, provides a robust mechanistic rationale for the outstanding reversible hydrogen storage performance of Ti–B_4_N_4_.

## 4. Conclusions

In summary, this work provides a comprehensive computational assessment of pristine and metal-decorated B_4_N_4_ nanocages as lightweight hydrogen storage materials. While the pristine nanocage exhibits excellent structural stability, its interaction with H_2_ remains dominated by weak van der Waals forces, resulting in negligible uptake under practical conditions. Lithium decoration enhances the adsorption strength of the first few hydrogen molecules through moderate polarization effects; however, the interaction rapidly weakens with increasing loading, limiting its reversible capacity. In contrast, titanium functionalization drastically reshapes the adsorption landscape. Ti induces strong polarization and enhances electrostatic and dispersion contributions, while maintaining a reversible, non-dissociative interaction with H_2_. Sequential adsorption analyses show that Ti–B_4_N_4_ achieves both high theoretical loading (20 H_2_) and remarkable retention at near-ambient conditions. Grand canonical thermodynamic evaluations confirm that Ti–B_4_N_4_ stores nearly its full loading at 30 bar and 298 K and releases a substantial fraction under mild desorption conditions, yielding an effective reversible capacity of 15.1 wt%, far exceeding the U.S. DOE target. The mechanistic insight provided by LMOEDA further clarifies that the superior performance of Ti–B_4_N_4_ arises from the cooperative strengthening of electrostatic, polarization, and dispersion interactions, which overcome the increased repulsion at high coverage and stabilize multiple H_2_ molecules. This multicomponent reinforcement is absent in pristine and Li-decorated systems and constitutes the key differentiating factor driving the exceptional storage properties of Ti-functionalized B_4_N_4_.

Overall, Ti–B_4_N_4_ emerges as a highly promising candidate for next-generation solid-state hydrogen storage. Its combination of low mass, strong yet reversible interactions, and favorable thermodynamic characteristics at near-ambient conditions underscores the potential of metal-decorated BN quantum dots as efficient hydrogen storage platforms. These findings provide a solid theoretical foundation for future experimental validation and for the rational design of advanced BN-based storage materials.

## Figures and Tables

**Table 1:** Geometrical and electronic properties of pristine and metal-decorated B_4_N_4_ nanocages.

Parameter	Pristine B_4_N_4_	Li-B_4_N_4_	Ti-B_4_N_4_
B–N (Å)	1.50	1.50–1.67	1.50–2.78
B–N–B (°)	75.10	74.34	88.54
N–B–N (°)	103.10	97.48	54.30
M–N_1_ (Å)	-	1.84	2.21
M–N_2_ (Å)	-	-	2.06
M–B_1_ (Å)	-	1.67	2.06
M–B_2_ (Å)	-	2.34	2.37
E_coh_ (eV)	−6.18	−5.75	−6.07
E_b_ (eV)	-	−2.30	−5.24
Metal charge (e)	-	+0.51	+0.82

**Table 2:** Calculated adsorption energy ${\bar{E}}_{\mathrm{ads}}$ in eV/H_2_ and the desorption energy E_des_ in eV, using the wB97x-3c/vDZP level of theory.

Pristine B_4_N_4_				Li–B_4_N_4_		Ti–B_4_N_4_		
**n (H_2_)**	${\bar{E}}_{\mathrm{ads}}$ (eV/H_2_)	E_des_ (eV)	n (H_2_)	${\bar{E}}_{\mathrm{ads}}$ (eV/H_2_)	E_des_ (eV)	n (H_2_)	${\bar{E}}_{\mathrm{ads}}$ (eV/H_2_)	E_des_ (eV)
1	−0.12	-	1	−0.15	-	1	−0.36	-
2	−0.10	−0.08	2	−0.15	−0.14	2	−0.31	−0.25
3	−0.10	−0.08	3	−0.14	−0.12	3	−0.23	−0.08
4	−0.08	−0.07	4	−0.13	−0.09	4	−0.22	−0.21
5	−0.07	−0.06	5	−0.11	−0.05	5	−0.19	−0.03
6	−0.07	−0.06	6	−0.10	−0.07	6	−0.17	−0.06
7	−0.07	−0.06	7	−0.10	−0.05	7	−0.15	−0.06
8	−0.07	−0.05	8	−0.09	−0.07	8	−0.14	−0.07
9	−0.06	−0.05	9	−0.09	−0.05	9	−0.13	−0.03
10	−0.06	−0.05	10	−0.08	−0.05	10	−0.12	−0.05
11	−0.06	−0.05	11	−0.08	−0.07	11	−0.12	−0.06
12	−0.06	−0.05	12	−0.08	−0.05	12	−0.11	−0.03
13	−0.06	−0.05	13	−0.08	−0.04	13	−0.10	−0.04
14	−0.06	−0.05	14	−0.07	−0.03	14	−0.10	−0.06
			15	−0.07	−0.06	15	−0.10	−0.03
			16	−0.07	−0.05	16	−0.10	−0.08
			18	−0.07	−0.05	17	−0.09	−0.05
						18	−0.09	−0.05
						19	−0.09	−0.04
						20	−0.09	−0.04

**Table 3:** Calculated thermodynamic and kinetic desorption parameters (T_D_ in K, τ) and reversible storage metrics (N_A_, N_D_, N_P_, C_E_) for pristine and metal-decorated B_4_N_4_ at selected pressures and temperatures.

System	Pristine B_4_N_4_	Li–B_4_N_4_	Ti–B_4_N_4_
T_D_ (1 bar)	115.1	140.6	287.7
T_D_ (3 bar)	130.9	160.0	327.3
T_D_ (30 bar)	184.1	224.9	400.14
T_D_ (100 bar)	233.6	285.6	484.1
τ (233 K)	0.21 ns	0.89 ns	0.30 ms
τ (298 K)	0.06 ns	0.18 ns	0.61 µs
τ (358 K)	0.03 ns	0.07 ns	0.06 µs
τ (400 K)	0.02 ns	0.04 ns	0.02 µs
C_T_ (wt%)	22.1	25.3	21.4
N_T_	14	18	20
N_A_	0.37	1.73	19.89
N_D_	0.01	0.03	6.80
N_P_	0.36	1.7	13.2
C_E_ (wt%)	0.8	3.1	15.1

**Table 4:** Comparative effective hydrogen storage capacities of pristine and decorated B_4_N_4_ nanocage and selected lightweight nanostructured materials.

Material	Metal Decoration	wt%	Ref.
2D C_2_N layer	Li	9	[46]
Graphene	Li	6–8	[11]
Defected graphene	Li, Mg	8–10	[48]
Carbon nanotubes	Ti	4	[30]
C_60_ fullerene	Li	7	[47]
Defected biphenylene	TM	10	[45]
Irida-graphene	Sc	21.6	[49]
R-graphyne-MOF	Li	11.9	[50]
IRMOF-16	Mg	5.8	[51]
IRMOF-10	Li	8.3	[52]
COF-1	Sc	5.23	[53]
COF-1	Li	7.7	[54]
Azatriphenylene-COF	Y	6.4	[55]
CTF-1	Zr	7.1	[56]
AzaCOF	Ti	9.3	[57]
C_8_ quantum dot	Mg	21.7	[58]
C_3_B_2_ quantum dot	Ti	20.10	[59]
Pristine B_4_N_4_	-	0.8	This work
B_4_N_4_	Li	3.1	This work
B_4_N_4_	Ti	15.1	This work

**Table 5:** Energy decomposition analysis of single and multiple hydrogen interactions with B_4_N_4_, Li-B_4_N_4_, and Ti-B_4_N_4_ systems.

Energy (eV)	H_2_/B_4_N_4_	H_2_/Li-B_4_N_4_	H_2_/Ti-B_4_N_4_	14H_2_/B_4_N_4_	18H_2_/Li-B_4_N_4_	20H_2_/Ti-B_4_N_4_
Electrostatic	−0.62	−0.10	−0.47	−1.37	−0.96	−1.96
Exchange	−1.06	−0.06	−0.60	−1.90	−0.79	−2.29
Polarization	−0.96	−0.08	−1.03	−1.37	−0.60	−2.12
Dispersion	−0.43	−0.08	−0.37	−1.92	−1.55	−2.71
Repulsion	2.93	0.22	1.60	5.90	2.76	6.83
Total	−0.13	−0.11	−0.87	−0.65	−1.14	−2.24

## Data Availability

The data supporting the findings of this study are available within the article and its Supplementary Information. Further inquiries can be directed to the corresponding author.

## Supplementary Materials

The following supporting information can be downloaded at: https://www.mdpi.com/article/10.3390/nano16090499/s1, Table S1: Mulliken atomic charges (e) for pristine and metal-decorated B_4_N_4_ nanocages; Table S2: HOMO, LUMO, and HOMO–LUMO gap (ΔEg) of pristine and metal-decorated B_4_N_4_ nanocages before and after H_2_ adsorption, calculated at the ωB97X-3c level.
