# Supplementary material for: Metal Decorated B4N4 Nanocages Quantum Dots for Hydrogen Storage: A Comprehensive Density Functional Theory Approach
Source: Nanomaterials (Basel). 2026 Apr 22;16(9):499. doi: 10.3390/nano16090499 (PMC13165286; doi:10.3390/nano16090499)
Supplement: Supplementary file 1 [file nanomaterials-16-00499-s001.zip › nanomaterials-4240238-supplementary.pdf]

Metal Decorated B_4N_4 Nanocages Quantum Dots for Hydrogen Storage: A Comprehensive Density Functional Theory Approach

Seyfeddine Rahali [1,*], Youghourta Belhocine [2], Ridha Ben Said [1], Yusuf Zuntu Abdullah [3,4], Tasneem I. Hussein [1] and Bakheit Mustafa [1]

[1] Department of Chemistry, College of Science, Qassim University, Buraydah 51452, Saudi Arabia; r.said@qu.edu.sa (R.B.S.)
[2] Laboratory of Catalysis, Bioprocess and Environment, Department of Process Engineering, Faculty of Technology, University of 20 August 1955, Skikda 21000, Algeria; y.belhocine@univ-skikda.dz
[3] Department of Physics, Aydin Adnan Menderes University, Aydin 09010, Turkey
[4] Department of Physics, Faculty of Science, Kaduna State University, PMB 2339 Kaduna, Nigeria
* Correspondence: s.rahali@qu.edu.sa

Table S1. Mulliken atomic charges (e) for pristine and metal-decorated B_4N_4 nanocages.

System	Atom	Charge (e)
Pristine B_4N_4	N (×4)	–0.235
	B (×4)	+0.235
Li–B_4N_4	N1	–0.411
	N2	–0.237
	N3	–0.291
	N4	–0.291
	B1	+0.217
	B2	+0.217
	B3	+0.115
	B4	+0.172
	Li	+0.5099
Ti–B_4N_4	N1	–0.206
	N2	–0.174
	N3	–0.586
	N4	–0.586
	B1	+0.252
	B2	+0.252
	B3	–0.053
	B4	+0.286
	Ti	+0.815

Table S2. HOMO, LUMO, and HOMO–LUMO gap (ΔEg) of pristine and metal-decorated B_4N_4 nanocages before and after H_2 adsorption, calculated at the ωB97X-3c level.

Nanocage	B_4N_4	Li-B_4N_4	Ti-B_4N_4	H_2/B_4N_4	H_2/Li-B_4N_4	H_2/Ti-B_4N_4
HOMO (eV)	-11.73	-7.36	-8.03	-11.74	-7.29	-8.06
LUMO (eV)	-1.83	-0.94	-0.17	-1.85	-0.89	-0.18
ΔE_g (eV)	9.90	6.42	7.86	9.89	6.40	7.88

Cartesian coordinates (in angstroms) performed at ωB97X-3c/vDZP level of theory

Pristine B_4N_4

N	0.13244732449621	-0.70057653600023	1.24853304439224
N	0.11653029420476	-0.93283188790749	-1.08771749858191
N	-1.28511207919775	0.64228467817360	-0.05518160567021
N	1.03612304233402	0.99112096821809	-0.10560833796275
B	-0.80666859126050	-0.77173934845280	0.08227453506669
B	1.00069102029611	-0.50000494025466	0.04290315218575
B	-0.09086530784232	0.72626466705458	0.84703209272439
B	-0.10314570303052	0.54548239916890	-0.97223538215420

Li-B_4N_4

N	-0.27009590037629	-0.64502751096874	1.26843988078958
N	0.19688789838752	-0.96722508100449	-1.05463206496050
N	-1.29950205403830	0.76127788804757	-0.37633437058391
N	1.03820717740914	0.96276205694366	0.06557908018539
B	-0.96384990828740	-0.69222815567413	-0.12562095020178
B	0.87940372675076	-0.53333679773369	0.22288986508765
B	-0.30162719036380	0.83618262485504	0.76063160758184
B	0.05894476583789	0.54136414225939	-1.01231224437946
Li	-0.51583901531953	-1.35973333672462	2.90958161648119

Ti-B_4N_4

N	-0.07505599893656	1.23744214891555	-1.62697266081066
N	0.11970723730921	-1.02399628410716	-2.01856787402498
N	-1.23406276135195	-0.68077555838655	-0.15576958546826
N	1.29509037732626	-0.47462979390977	-0.08510375400603
B	-0.89335503717734	-0.10411417611052	-1.46306319441635
B	0.93848413882429	0.04478562797950	-1.41217537649050
B	-0.16317773230082	1.86993826480153	-0.43165601998618
B	0.09606373934696	-1.23973044190229	-0.54331481352075
Ti	-0.08369396304005	0.37108021271972	1.19223696872373

H_2/ B_4N_4

N	0.60977000755668	-0.52248332301226	1.17605045700698
N	-0.20608904939177	-1.08148135576743	-0.95586726993049
N	-1.42376405299116	0.38510693353614	0.42646339513931
N	0.60737459681557	1.08608099227228	-0.53713333272952
B	-0.65054050095777	-0.89956976064092	0.46444398873719
B	0.91819321467572	-0.35736100244678	-0.27872366290548
B	-0.02160879336610	0.77647988545440	0.78836152317549
B	-0.67617771375314	0.36031843234667	-0.89677122740720
H	-1.87861675567786	1.01127824724265	-2.01876476072588
H	-1.27727170291016	0.91834878101524	-2.47630801036041

H_2/Li-B_4N_4

N	0.10903842753922	-0.81570743216094	1.26944059552792
N	0.40677841847937	-0.95424432952752	-1.10364245787417
N	-1.42919389149491	0.33592415503871	-0.19977913809073
N	0.78539465524948	1.07235360294006	0.03482378286265
B	-0.68569071770280	-1.14209381010544	-0.02018514646674
B	1.02718990546663	-0.40413573856244	0.15139028966409
B	-0.35340845177065	0.54376484455788	0.84647683763107
B	-0.12582431503429	0.43787286087115	-0.96607409991992
Li	-2.96440308695067	-0.67423609400008	-0.34799031040724
H	-4.93476962949889	0.15831102297174	-0.14590724868174
H	-4.45976848428248	0.73122624797689	-0.06716893424519

H_2/Ti-B_4N_4

N	0.44371172164337	0.89583356209619	-1.56984585801205
N	-0.71993844161370	-0.99005309060096	-2.20112247629243
N	-1.65999340368757	-0.12537124051600	-0.25319522888563
N	0.50139308187077	-1.45201198989781	-0.27184530389692
B	-1.01291246242208	0.28163379017024	-1.50904263960260
B	0.55501135446210	-0.68147431480697	-1.52265912196158
B	0.69630567412908	1.29536517626653	-0.30007457714743
B	-0.90531793366993	-1.31146848584643	-0.75757781957056
Ti	-0.15976458321238	-0.12815510710665	1.18231290781560
H	0.53544223011218	1.00290234930809	2.99804325757699
H	0.18131987238819	0.42729590093378	3.35331914997660

14H_2/B_4N_4

N	0.65740650253859	-0.62731216038654	1.29032995100918
N	-0.61201447424721	-0.80238806164765	-0.68517582740634
N	-1.22761823372373	0.74633036655465	0.97566479091295
N	0.71768836933170	1.10297080694272	-0.30352275462190
B	-0.76375772781789	-0.67124423382487	0.80394266375199
B	0.75714986345390	-0.39951188533194	-0.19652150640000
B	0.27079989137308	0.81600127279150	1.10612820110121
B	-0.72332924941349	0.68069093381361	-0.44232736409682
H	-2.07704329757189	1.60770006010956	-1.67779575814595
H	-1.42928142270528	1.89919434288026	-1.92401307264719
H	2.49923723360302	-1.28114560209981	-0.78347359476265
H	2.07417336908194	-1.39574125370467	-1.39345320856130
H	1.21174037096083	2.27090987340658	2.26097949471084
H	0.70411651347078	2.02989518772826	2.75891892935004
H	-1.71913255693090	-2.39637320944453	1.88266726783195
H	-2.32685375364891	-1.98688132796270	1.73457217168425
H	-3.91692202755398	-0.20345916131747	-0.53441522114821
H	-3.52538694499402	-0.66240517749125	-0.96976670898668
H	3.62684222280105	0.50024842663841	1.21123630839502
H	3.50724093485817	0.97479606340043	0.65082906700845
H	0.83308498358864	0.52701346449692	-3.37814613564483
H	0.37209807626928	-0.05718907411668	-3.39282790382059
H	-0.23853397395075	-0.32598559400058	3.99457287986200
H	-0.81054740286718	0.06957746539292	4.25910775203154
H	0.80149857616978	-3.70105084095202	0.32812912044810
H	0.30013475144437	-3.60859406259756	-0.21401087418753
H	-0.49915022072591	3.94872047565534	0.20490323048899
H	-1.06145995510133	3.75549238618708	0.65226164529268
H	-2.06789975591620	2.41139126223647	3.00020211483656
H	-2.08442796043701	2.90259873457219	3.56017920434369
H	3.44110268501473	-2.63820793879282	1.73612448653242
H	2.81944195369187	-2.23548624094434	1.81661522051987
H	0.80504861111910	3.65479316225006	-2.62971460149120

H	0.95393967398708	3.14338044070144	-2.10862366488269
H	-0.77156611429853	-2.63979517516128	-2.68727169370105
H	-0.75453898085371	-3.17944761598171	-3.20140267960683

$18H_2$/Li-B_4N_4

N	0.897124	-0.683518	0.812102
N	0.353098	1.092653	-0.692437
N	-1.363075	0.101927	0.651627
N	0.316189	1.471816	1.663460
B	-0.130751	-0.266499	-0.224556
B	1.133587	0.798353	0.576457
B	-0.158613	0.033939	1.629641
B	-0.584435	1.424391	0.447907
Li	-3.116196	-0.499103	0.706002
H	-4.532974	1.265839	0.616739
H	-3.844099	1.551469	0.665947
H	-2.340066	-2.513337	0.791669
H	-3.055530	-2.724939	0.843475
H	-3.088661	-0.509404	-1.472167
H	-3.805081	-0.720863	-1.435364
H	-3.053358	-0.283842	2.880291
H	-3.723215	-0.615593	2.895152
H	-5.348006	-1.564823	0.498308
H	-5.499417	-1.106096	1.065567
H	0.372359	-3.342900	0.821689
H	0.032445	-4.006384	0.810983
H	-0.863228	0.387890	-3.154838
H	-1.334086	0.104446	-3.657974
H	-2.080817	2.543708	2.817101
H	-2.787781	2.681422	3.007467
H	-2.970384	2.646934	-1.823276
H	-2.250831	2.456756	-1.796377
H	-0.782238	-2.823210	-1.772982
H	-1.360586	-2.933189	-2.228523
H	-0.659051	-2.446613	3.289068
H	-1.253642	-2.546429	3.725743
H	0.393423	3.163950	-2.473701
H	0.287111	3.738310	-2.936774
H	0.584379	4.097131	3.859883
H	0.611532	3.520791	3.387566
H	2.223296	-2.132367	2.725967
H	2.454664	-2.499422	3.331687
H	-0.879719	0.531714	5.054137
H	-0.521051	0.880331	4.502644
H	2.482707	0.526029	-3.413150
H	2.030515	0.733073	-2.858323
H	-0.786653	4.389051	0.861095
H	-1.140597	4.684405	0.276840
H	2.177276	-2.261914	-1.138034
H	2.331277	-2.605836	-1.780343

$20H_2$/Ti-B_4N_4

N	1.575607	1.508966	-1.003284
N	0.915679	-0.680576	-1.279596
N	-0.879114	0.559167	-0.463993
N	1.064361	-0.215778	0.996641
B	0.286973	0.654722	-1.348139
B	1.692989	0.094709	-0.292614
B	1.140734	2.305219	-0.000188
B	0.061228	-0.530258	-0.064818
Ti	-0.304737	1.369476	1.436430
H	0.600081	-1.896443	3.086156

H	0.283262	-2.201604	3.688850
H	-0.281043	3.388467	2.473971
H	-0.768152	3.055885	2.952986
H	-1.375753	-0.361792	2.602027
H	-1.960149	-0.109157	2.198265
H	-2.378375	2.236949	0.708927
H	-2.484002	2.491283	1.409848
H	1.083675	0.885783	3.288784
H	0.595197	1.272655	3.711716
H	-2.954361	1.848398	4.428873
H	-3.252283	2.499212	4.230421
H	-1.107740	-2.843204	0.854295
H	-1.663958	-2.593640	0.426077
H	-3.407425	-0.216315	0.006508
H	-4.038480	-0.386311	0.366619
H	-0.661467	5.072049	0.395148
H	-1.054566	5.646871	0.658123
H	4.233130	-0.105743	-0.555952
H	4.135038	-0.435929	0.105062
H	-1.567806	3.547209	-2.056049
H	-1.702449	2.851238	-1.827141
H	1.725546	3.580348	-3.059734
H	1.549141	4.228574	-3.380421
H	3.462307	0.691580	-3.876388
H	3.075206	0.903777	-3.276573
H	3.648377	-0.668768	3.185537
H	3.082718	-0.701351	2.700161
H	0.055751	0.845929	-3.907791
H	-0.302503	1.493076	-3.820088
H	0.277360	-4.043035	-2.170043
H	0.459938	-3.331865	-2.044794
H	2.273350	-3.545706	0.778187
H	2.043564	-2.845313	0.889126
H	-2.345977	-1.590117	-2.880130
H	-2.124900	-1.116912	-2.348541
H	2.129294	4.963228	2.903367
H	2.117050	4.493541	2.326635
H	4.811587	3.126378	-1.068045
H	4.188476	2.800394	-1.311638
